# Ondasetron versus haloperidol for the treatment of postcardiotomy delirium: a prospective, randomized, double-blinded study

**DOI:** 10.1186/1749-8090-7-25

**Published:** 2012-03-21

**Authors:** Georgios I Tagarakis, Christos Voucharas, Fani Tsolaki, Marios E Daskalopoulos, Vassilios Papaliagkas, Charalampos Parisis, Eleni Gogaki, Ιlias Tsagalas, Ιlias Sataitidis, Magda Tsolaki, Nikolaos B Tsilimingas

**Affiliations:** 1 Department of Cardiovascular and Thoracic Surgery, University Hospital of Larissa, Thessaly, Greece; 2 3rd Department of Neurology, Aristotle University of Thessaloniki, Thessaloniki, Greece; 3 Anaximandrou 24, 54250 Thessaloniki, Greece

## Abstract

**Background:**

To investigate the controlling efficacy of ondasetron and haloperidol in regard to the postcardiotomy delirium.

**Methods:**

We included in this prospective, randomized, double-blinded study 80 patients who developed delirium after heart surgery with the application of heart lung-machine. The patients were divided into two, equally-sized groups, which on detection of delirium received ondasetron 8 mg iv or haloperidol 5 mg iv respectively. The statistical analysis compared the baseline and demographic characteristics of the two groups (age, gender, comorbidities, years of education, type of surgery etc.).

**Results:**

Both ondasetron and haloperidol had very good delirium controlling effects, without statistically significant differences.

**Discussion-Conclusions:**

Ondasetron and haloperidol are efficient agents as far as the treatment of postcardiotomy delirium is concerned. As, in addition, ondasetron bares milder side-effects, we believe this could be the agent of choice in patients developing postcardiotomy delirium in the future.

## Background

Neuropsychiatric complications belong to the most common and feared ones after heart surgery. They include a variety of nosological entities, such as ischemic/embolic stroke, transient ischemic attack, postoperative cognitive decline and postoperative delirium [1-3]; in the broad sense, ocular neurosensory disorders ranging from blurred vision to amaurosis fugax also fall to the same category [4]. The pathophysiological background of these disorders includes low cerebral perfusion pressure, especially during extracorporeal circulation, various sorts of embolism (through clot, atheromatic or gas particles) and the influence of the anesthetic drugs regimen. Predisposing/precipitating factors regarding the patient's medical condition and baseline characteristics include previous stroke, occlusive carotid artery disease, diabetes mellitus, atrial fibrillation, peripheral occlusive arteriopathy and low educational niveau [1-3,5]. Procedure-related conditions enhancing the danger for the development of these complications include urgent surgery, long duration of surgery, long duration of extracorporeal circulation, long aortic cross-clamp time and type of surgery (on-pump) [1-3,5,6].

Postoperative delirium can be an extremely unpleasant situation because of the following characteristics: i) it occurs at a high frequency, ranging from 8% to 32% of heart-operated patients [1-3,7,8] ii) it usually appears during the first hours/days after extubation and totally disorganizes the patient right when he needs to be focused on his rehabilitation, iii) it can be accompanied by serious injuries of the patient (e.g. falling from bed or violent removal of central venous/arterial lines or epicardial electrodes) iv) it can be the cause of life-threatening arrhythmias or unexpected elevation of blood pressure v) there is no special and generally accepted algorithm for its treatment except for general symptomatic measures (fixation of the patient) or application of sedative/psychotropic drugs with possible subsequent occurrence of related complications. Herein, we are presenting our own experience with a prospective, randomized, double blinded study comparing ondasetron and haloperidol in regard to their delirium-controlling efficacy after heart surgery.

## Ondasetron

**Figure 1:**
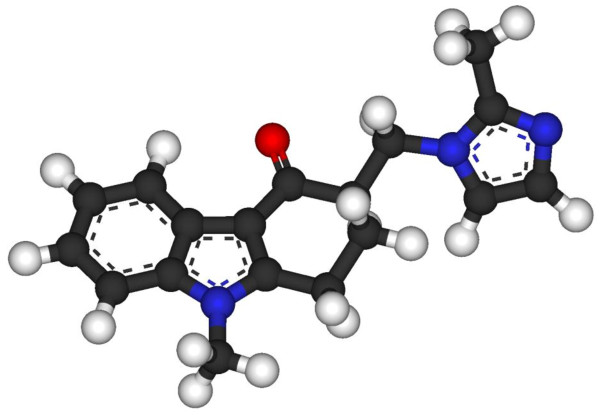
**Chemical structure of ondasetron**.

Ondasetron is a serotonin 5HT-3 antagonist, mainly used as an agent against nausea and vomiting; it is usually applied to patients after chemo- or radiotherapy or after surgery [8]. Many authors believe that the drug can be also indicated in other morbid situations such as schizophrenia, Parkinson's disease and alcohol addiction with beneficial psychotropic effects for the related patients. It is generally considered as a safe medicine with the most known side-effects being constipation, dizziness and light headache. Ondasetron is metabolized hepatically, excreted renally and has a half-life of 5.7 h. Its chemical structure is shown on Figure 1.

In 2000 [9,10], Bayindir et al., published the results of an interesting study which reported a good controlling effect of intravenously administered ondasetron (8 mg) in patients with postcardiotomy delirium. These results have never been confirmed by a larger study; furthermore, no direct comparison of ondasetron with other pharmaceutical agents has ever been performed by the aforementioned or another research.

## Haloperidol

**Figure 2:**
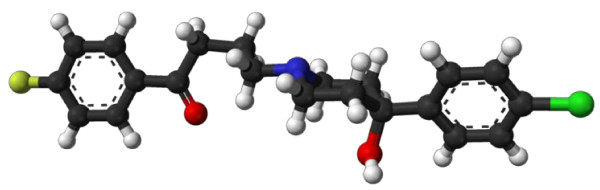
**Chemical structure of haloperidol**.

Haloperidol is a potent neuroleptic and psychotropic agent belonging to the group of butyrophenones. It mediates its action through blockade of dopaminergic receptors in the mesocortex and limbic system of the brain. Secondarily, it also has antimuscarinic and anticholinergic properties. Haloperidol has a significant efficacy against delirium and hallucinations, as well as anti-nausea and antivomiting properties. It is most commonly used in schizophrenia, acute psychotic states and delirium. Its most known side-efffects are the extrapyramidal ones (akathisia, tardive dyskinesia, muscle stiffness). Haloperidol is hepatically metabolised, biliary-renally excreted and has a half-life of 12-36 hours. Its chemical structure is shown on Figure 2.

## Methods

After the ethics committee approval and the patients'/relatives' informed consent we recruited for the study 80 consecutive patients who developed postoperative delirium after on-pump heart surgery (coronary artery bypass graft surgery, aortic valve replacement surgery, mitral valve surgery or combined procedures). The patients were gathered during a 3-year period (2008-2010) out of a total of 600 patients operated during the same period in our department. Exclusion criterion for the study was the history of severe psychiatric disease (schizophrenia, bipolar disorders). For the detection and evaluation of postoperative delirium we applied a practical scale, similar to that used by Bayindir et al. [8]. This 4-point scale was rated as follows: 0-normal, 1-patient with restlessness and mild confusion but cooperative, 2-patient disorientated but cooperative, memory gaps, 3- patient disorientated and uncooperative with augmented mobility that could put him to danger, 4-patient totally disorientated, violent and aggressive, presence of hallucinations. We divided our sample patients into two groups: i) the ondasetron group, whose members were given 8 mg ondasetron iv on detection of delirium, ii) the haloperidol group, whose patients were given 5 mg haloperidol iv. The two substances were administered at a random, alternate (one but one) order. All patients were evaluated before and 10 min after the injection with the aforementioned scale. The idea for a third, placebo control group was abolished as we considered dangerous and unethical to leave these patients totally untreated for their delirious condition.

### Statistical analysis

For the statistical analysis, the χ2 criterion was used for categorical parameters and the t-student unpaired for the continuous ones. The t-student (paired) criterion was applied to check for test scoring differences prior and after the drugs administration within each group. The ANOVA continuous measurements and the comparison of percentages were used to check for differences in the test scoring improvement between the two groups. The Kolmogorov-Smirnov test was used to check for normal distribution of the values. The level of statistical significance was set at a value for *p *< 0.05.

## Results

There were no significant differences regarding the baseline characteristics between the two groups. We noted a statistically significant improvement in the test score rating after the administration of both ondasetron (from 3.1 to 1.2, percentage improvement 61.29%, *p *< 0.01) and haloperidol (from 3.1 to 1.3, ± percentage improvement 58.064%, *p *< 0.01). No statistically significant differences were found as far as the post-application improvement between the two groups is concerned. 7 patients in the ondasetron group and 6 in the haloperidol group remained delirious after the pharmaceutical application, had to be fixated and returned to calmness after falling asleep in a period ranging from 2-5 hour. The aforementioned data are depicted in detail in Tables 1 and 2.

**Table 1:** Baseline characteristics of study patients

	Ondasetron group	Haloperidol group	Statistical Significance
**Sample Size**	40	40	n.s
**Gender (Male)**	27	26	n.s
**Age**	70.1 ± 9.3	70.9 ± 9.9	n.s
**Type of surgery: CABG**	34	35	n.s
**Type of surgery: Aortic valve**	2	1	*P *< 0.05
**Replacement**			
**Type of surgery CABG and aortic**	2	2	n.s
**valve replacement combined**			
**Mitral valve surgery**	1	1	n.s
**Aortic Surgery**	1	1	n.s
**Years of Education**	7.3 ± 0.7	7.8 ± 0.7	n.s.

The table shows baseline characteristics for both groups of patients. The χ2 criterion was used for the comparison of categorical parameters, the t-student unpaired for the comparison of continuous ones. Level of statistical significance was set at a value of *p *< 0.05.

**Table 2:** Efficacy of ondasetron and haloperidol

**Ondasetron group mean rating prior to application**	**Ondasetron group mean rating after application**	**Statistical Significance**
3.1 ± 0.4	1.2 ± 0.1	*p *< 0.01
**Haloperidol group mean rating prior to application**	**Haloperidol group mean rating after application**	**Statistical Significance**
3.1 ± 0.4	1.3 ± 0.1	*p *< 0.01
**Ondasetron effect on rating(improvement in percentage)**	**Haloperidol effect on rating(improvement in percentage)**	**Statistical significance**
61.29%	58.064%	n.s
**Ondasetron patients that remained delirious**, n = 7	**Haloperidol patients that remained delirious**, n = 6	n.s

The table shows pre-and post application test score ratings for both ondasetron and haloperidol groups; in addition, the comparison in improvement percentage rates between the two groups with statistical significance is also depicted. The t-student (paired) criterion was used for the detection of pre-and postapplication test scoring differences within each group. The ANOVA continuous measurements and the comparison of percentages methods were applied for the comparison of test scoring improvement between the two groups. Level of statistical significance was set at a value for *p *< 0.05.

## Discussion

Postoperative delirium remains a major problem for patients undergoing any kind of heart surgery. Besides their heart condition, these patients are usually of an increased age and carry multiple morbid conditions, such as diabetes mellitus, arterial hypertension, previous stroke, occlusive carotid artery disease etc. The occurence of delirium hinders their normal course of recovery and rehabilitation, endangers their life through various injuries, prolongs their ICU and hospital stay, the number of rehospitalizations, and finally, the total hospitalization expenditure [11]. For these reasons we planned and performed this study in an effort to establish a safe and standard pharmacological approach for the treatment of postoperative delirium.

We included in our comparative analysis two well-known agents: ondasetron, with good anti-nausea and antivomiting properties and haloperidol, a widely used neuroleptic and psychotropic agent recruited against acute psychotic conditions and hallucinations. Our study concluded to an excellent and equal delirium-controlling effect of the two drugs. Although this has been known for haloperidol, it consists practically new knowledge for ondasetron, as only one study (by Bayindir et al.) in the past has reached the same result; this study had the disadvantage of being based on a small sample of patients and of missing comparative information with another, already used delirium-controlling agent (as for example in our study haloperidol). If we expand our comparison between ondasetron and haloperidol to other parameters, we can say that they both are low-cost agents, but ondasetron is safer and with milder side-effects. For these reasons we believe that ondasetron, although lacking an official indication, is the agent of choice for postcardiotomy delirium, as it is efficacious, inexpensive and safe. Further and larger studies are needed to support our findings, but also to expand ondasetron's indications not only to postcardiotomy delirium, but to other kinds of delirium as well.

## Conflicts of interests

The authors declare that they have no competing interests.

## Authors' contributions

GT is the author of the paper. CV helped in the data and literature research. FT performed literature research and checked the patients' ocular disorders. MD performed linguistic control. VP checked the paper and performed literature research. CP was the attending cardiologist. EG checked the paper and performed linguistic control. IT checked the paper. IS checked the paper. MT checked the paper. NT was head of the department and supervised the study. All authors read and approved the final manuscript.
